# Medical News and Miscellany

**Published:** 1893-12

**Authors:** 


### Medical News and Miscellany.

---

**Subscribers**, in remitting for renewal of their subscription to the TEXAS MEDICAL JOURNAL, declare that "it is the onliest" medical journal in the State. Subscribe now for 1894.

---

**The Weekly Medical Review** is the first in the field we believe, with its "visiting list for 1894." Thanks for a copy. This annual is now as standard as a directory or a dictionary, and is well known to every doctor. It is a handy pocket companion. Price as usual, Perpetual, 75 cents. Address, J. H. Chambers & Co., St. Louis.

**Change the Name.**—The next meeting of the Tri-State Medical Society will be held in Atlanta, Ga., 2d Tuesday in October, 1894, on which occasion it will be proposed to change the name of the society to the South-Easthern Medical Society. This will embrace all the territory east of the Mississippi and south of the Ohio. Dr. Frank Trester Smith, of Chattanooga, is Secretary. The reason for this change is that there are two or three societies bearing the name "Tri-State."

---

**The South-Western Cocker's Journal,** edited and published by F. V. Daniel, Esq., at San Antonio, Tex. Monthly, $2 a year. We have received No. 1, Vol. 1 of the above sprightly publication and are much pleased with it. It is "devoted to the Cocker, the Field and the Turf" and will contain matters of interest to all lovers of sport. A prominent feature will be all that pertains to the breeding of game cocks. The publisher is a well known fancier and owns the finest strain of game birds in the State.

---

**The Next International Medical Congress** will, according to official announcement of Dr. A. Jacobi, chairman of the American National Committee of the International Medical Congress, be held at Rome, from March 29th to April 5th, 1894. The meeting which was to have taken place September 24, 1893, was postponed on account of cholera prevailing in Italy. Instructions, and documents relating to the journey, etc., are promised for the near future. Address Dr. A. Jacobi, 110 W. Sixth street, N. Y.

---

**Dr. Bennett,** well known to all our readers, met with a painful, though not serious accident on the afternoon of 2d inst. As he drove up to his office, his horse, a new purchase, shied at the pretty stair carpet just put down on the steps (it is red-striped —and hurt his eyes, may be), turned suddenly and tilted the buggy to such an angle that it must have upset, had the doctor not jumped out. As he did so, he slipped on the wet flag-stones, and fell heavily. His left shoulder was painfully strained and bruised, but no bones were broken.

---

**Another Journal.**—Dr. Jos. M. Matthews has announced that he will issue a quarterly journal, called *Matthews' Medical Quarterly*, beginning with January 1st, 1894. It is to be devoted to Diseases of the Rectum and Gastro-Intestinal diseases, Gastro-intestinal Surgery, and will contain from one hundred and fifty to two hundred pages. Price not stated. Dr. Matthews, as is well known, has given much attention to the subject to which his journal will be devoted, and will no doubt make it a most interesting and valuable addition to the list of medical periodicals.

---

**Wanted**—To learn the present address of the New York & London Chemical Company, lately doing business at 64 & 68 Broad street, New York. Previous to that time they were in the Morris Building. The JOURNAL is carrying their full page advertisement of Lithia Tablets; but letters to address given us do not fetch a reply, and our *drafts* are returned endorsed "not found." Several contemporaries "in the same boat" with yours truly are anxiously asking us "what do you know of the N. Y. & London Chemical Co.?" and one fellow suggests that they have gone to work the London end may be, perhaps; eh? We only want to know, you know, "where they are *at!*"

---

**Female Physicians in Turkey.**—Information has been received from Constantinople that our minister, Hon. A. W. Terrell, has gained a noble victory in diplomacy. The laws in Turkey do not recognize female physicians, but notwithstanding they have increased rapidly in that country, and a large number of them are composed of American missionaries. They have been embarrassed by the fact that they were unable to obtain Turkish diplomas, and the various ambassadors and foreign representatives have been very earnest in their efforts to secure for women the same rights to practice the profession of medicine as those enjoyed by men, and they have invariably failed, but, at last, under the persistent aud intelligent advocacy of our minister, Judge Terrell, Turkish conservatism has yielded and he has secured privileges for women practitioners of medicine which neither Russian, French, British nor German ambassadors could gain. Judge Terrell displays his gallantry and devotion to the gentler sex both at home and abroad.—*Statesman.*

---

**It is due Dr. Jno. B. Roberts**—(apropos of "What a Fish a Frog is" in our last), to say that the JOURNAL has received a letter from Dr. S. Solis Cohen saying that we do Dr. Roberts injustice, in classing him as of those who would "abolish the code in order that homeopathic patronage might flow as grist to the specialty mill." "Had he been," Dr. Cohen says, "he could never have been elected to the presidency of the Philadelphia County Medical Society." The JOURNAL certainly has no wish to do any one an injustice; and as Dr. Cohen says that Dr. Roberts is, after all, a good sort of a fellow, or words about to that import, we ask his pardon. Only, we were at a loss to understand upon what grounds he could urge a "homologating" of all sorts and conditions, a swallowing up of the homeopathic element, all but their name, which, we understood, they were to still be permitted to wag; something we suppose as it is said the Alaska fox does, as a bait to catch gudgeons. Dr. Roberts is a gentleman, but he laid himself open to ridicule, and deserves all he got probably.

---

**That Joke.**—In our October issue we said:

"THE MEDICAL FORTNIGHTLY.—'The Happy Medium' offers a prize for every error found in its advertising pages, and actually awarded a prize to a subscriber who pointed out that 'substitution' ought to have been spelled 'substitusion.' Good joke on Webster."

Brother Lewis, of the *Fortnightly*, felt worried at being thus caught napping, and tries to get out of it as follows:

"No, brother Daniel, the *joke* is on *you*. The error pointed out by Dr. Alvord was the careless 'substitution' of a capital 'C' for an 'S' after the form had gone to press. Compare issue of September 15, Leland Miller's card, and next time 'look before you leap,' doctor."

If any reader of the *Fortnightly* will refer to the October 1st number of that publication—(not Sept. 15th) he wlll find, on page 232, the following, verbatim.—(We always look before we leap:)

REWARD FOR ERRORS.

ST. LOUIS, MO., Sept. 16, 1893.

DEAR DOCTOR [LEWIS]:—In Leland Miller's advertisement oughtn't "substitution" to be spelled with an "s"?

GEO. E. ALVORD, M. D.

[REPLY.]

*Prize Winner No. 1.*—Dr. Geo. E. Alvord, St. Louis, wins a copy of the Missouri State Medical Directory, for being the first to discover the typographical error in Leland Miller's card.

Good joke on Snyder. Poor Webster. (But he's dead now.) A word spelled correctly in the *Fortnightly* is pointed out as a "typographical error" and rewarded. Go up head, brother *Fortnightly*.

---

**Extremes in the Death-Rate of Typhoid.**—"Dr. Benjamin also remarked that the treatment of typhoid fever had improved or the character of the disease had become milder. His attention had been arrested by the report from the Cooper Hospital of Camden, where during the past two years the mortality from typhoid fever had been reduced to 2 per cent. One of the fatal cases was a man who undoubtedly would have gotten well had he not disobeyed orders and got out of bed when known to have a weak heart. He died snddenly of heart failure. When looking up the death rate from typhoid he found that in one hospital in Philadelphia it was 6 per cent, and the treatment employed, said to be the best, was by the cold pack. In the Cooper Hospital they had not used the cold pack, but had employed the antiseptic treatment of disinfecting the bowels. In the Pennsylvania Hospital the death rate from typhoid was 9 per cent last year. He found to his great astonishment that the Philadelphia Homeopathic Hospital (Hahnemann) reported a death rate from this disease of 24 per cent the last two years, 1891 and 1892.

"The location of their hospital was one of the best (and under the treatment of their leading professors) and he was unable to understand why they should have a death rate 1200 per cent higher than others, 100 per cent higher than the most unfavorable statistics in Philadelphia under the "old school" system, yet the homeopathics laid special claim to superiority in fevers, if in anything. The epidemic had been the same, the circumstances the same (except of course the treatment). No explanation had been offered, notwithstanding the daily press had mentioned the subject."—*From the Transactions of the Medical Society of New Jersey*, 1893; pages 36-37.

---

ERRATA.—In the Doctor Roberts paragraph the printer left off my Alaska fox's *tail*, and makes him wag a *name*, like a homeopath does. Good joke on us.

---

### THE EVILS OF SUBSTITUTION.

BY CYRUS EDSON, M. D.,
Commissioner of Health of New York City and State; President of the Board of Pharmacy of the City and County of New York.

The term "substitution," in its commercial sense, is the perpetration of a fraud by the seller upon the buyer, the former selling the latter something different from the article demanded, under the same name. This fraud is really but another phase of commercial adulteration, and in the practice of pharmacy its evils are as insidious and harmful as those of any crime committed by man. These evils are both direct and remote in their effects. They injure, first, the patient; second, the physician; third, the manufacturer. From the standpoint of the patient, the evil affects him directly and indirectly. The dishonest pharmacist has, of course, palmed off on his unsuspecting customer a cheaper preparation than that ordered by the prescriber, because the motive for the crime is, in ninety-nine cases out of a hundred, a mercenary one. The result to the patient from the inhibition of the substituted article may be one of the following: first, no therapeutic action; second, therapeutic action of less potency; third, therapeutic action of greater potency; fourth, therapeutic action of different character than aimed at by the prescriber. It needs no argument to prove that any of these four results would, under certain conditions, be likely to be disastrous to the patient.

The pharmacist is the responsible and trusted dispenser of the physician's order, and when he acts differently than ordered by the doctor, he snips at the threads of fate, possibly without the slightest idea of what will result from the snipping. Then he is no better than a man who fires a bullet among a crowd of people. The result in either case may be manslaughter. Let us take a less extreme view of the crime from the patient's standpoint. The latter fails to get benefit from his medicine, and, as a result, loses time and money. He was cheated when he bought the preparation. Now, indirectly, he lost the fee he paid the physician, and last, but not least, he has lost confidence in his doctor.

From the standpoint of the physician, the evils of substitution have a wider range in their effect than on the individual patient. Medicine has been said to be an inexact science. The reason of this is because it is very difficult to ascribe a given effect to a certain cause. In other words, so many causes operate to produce a given effect in the human economy, that it is difficult to ascertain and fix upon a definite cause. Modern therapeutics is the outcome of the physician's observations and experience of the effect of drugs upon the human system. It is a science to which every physician contributes his mite or his much, according to his ability and his opportunity.

The pharmacist who substitutes, leads physicians astray. By presenting false premises to the latter, the former causes him to make erroneous deductions. The entire medical profession may thus feel the result of a single instance of substitution, and numerous other invalids suffer on account of the errors following faulty experience in the case of the physician treating a single patient who is the victim of the fraud in question.

I have already spoken of the loss of confidence in his physician on the part of the victimized patient. This has not only a direct effect upon the invalid, because confidence in his doctor's efforts are, to a great extent, essential to the latter's success in the treatment of of the case, but it may also cause the dismissal of the physician, and his loss of what perhaps would have been a lucrative practice. In this country, physicians have the reputation of being practical. They are the best practitioners in the world. In other countries, medical men are deeper students and better theorists, but here, we pride ourselves on the results we obtain in curing disease. The reason for this is because we strive less for the honor and glory than we do for the almighty dollar. We must give our patients the worth of their money, and we know that we will not be tolerated unless we do. Our patients are quick to discover mistakes, and they are laid at the door of the physician rather than at that of the pharmacist. If this was not the case, the subject of substitution would not be worth consideration, for it would be a rarely committed crime.

The question of injury to the manufacturer is a very important phase of the matter, for, rather singularly, the remedy for the great evil must spring mainly from this source. This is not so strange after all, when we come to think of it, for here we find the effects of the evils of substitution so direct and so distinctly felt that interest is natural. Nothing causes men more concern than pecuniary loss. Cause and effect are here so closely associated that a hue and cry at once follows. The manufacturer invests large sums in producing a reliable preparation; he spends more in bringing it before the medical profession. The latter find it worthy of use, and patronize it until the weeds of substitution check its growth. The way these weeds act, after what I have said is obvious. For example, some pharmacist substitutes an inferior mixture or drug, in the preparation of the phytician's prescription; the effect of the medicine on his patient is nil. The disappointed doctor heralds the fact to his brethren. Such news travels faster than any favorable comments, and undoes in a short time that which the manufacturer has taken months, or perhaps years, to accomplish. Great injury is in consequence done to a deserving business.

Then again, the evil is a widespread one, and the same substitution in a good preparation is very large, and directly affects its sale. I know of no other crime that tends so much to destroy one's faith in man's goodness as substitution. For the sake of insignificant profit, the dishonest pharmacist deliberately cheats, and perhaps destroys his fellow man. I can only account for the practice by assuming that the perpetrator in some way persuades himself that he is doing no harm, that he is selling something "just as good," that he holds the judgment and knowledge of the physician in small repute, and that he feels perfectly competent to act in the premises. It is a curious psychological fact that it is the easiest thing in the world for a man engaged in a nefarious trade to persuade himself that he is doing no harm, so long as he is making money by his acts.

To correct the practice of substitution does not seem to me a difficult matter. A few years ago the adulteration of food products was a very serious fraud. Confectionery, for example, was greatly adulterated at that time. The exposure of the practice by the Health department of New York City so injured the confectionery business that the reputable manufacturers banded together in an Anti-Adulteration League. Not only did the Health Department cause the formation of the league in the way I have described, but the unfair competition engendered by adulteration also had its effect in forcing honest manufacturers to protect themselves. This league made it its business to run down and punish all persons who adulterated their wares. The result was that in a short time adulteration ceased, and to-day it is impossible to find any adulterated candy offered for sale. Another instance of manufacturers banding together for mutual protection is offered by the Jewelers' Protective Association. This body pursues like an avenging Nemesis any one who robs or cheats its members. Let the manufacturers of pharmaceutical preparations who suffer from the evils of substitution form a like union, and charge its agents with the duty of bringing to justice the perpetrators of the fraud of substitution. The penal code and the pharmacy act both afford excellent laws for the punishment of these criminals. The Board of Pharmacy is not sufficiently equipped to enforce the provisions of the law to this end, and the Health Department is too busily engaged in fighting disease to cope with the evil. The formation of such a union as I have indicated, however, and the punishment of a few offenders, would soon stop the practice. The mere publication of a few instances of fraud, giving the names and addresses of the dishonest pharmacists, would go far towards suppressing substitution, for the public is quick to discover and shun the druggist who is considered unreliable and unscrupulous.—*Doctor of Hygiene.*

